# Supplementary material for: Traumatic brain injury promotes neurogenesis at the cost of astrogliogenesis in the adult hippocampus of male mice
Source: Nat Commun. 2024 Jun 18;15:5222. doi: 10.1038/s41467-024-49299-6 (PMC11189490; doi:10.1038/s41467-024-49299-6)
Supplement: Supplementary file 1 — Supplementary information [file 41467_2024_49299_MOESM1_ESM.pdf]

Supplementary information file associated to:

Traumatic brain injury promotes neurogenesis at the cost of astrogliogenesis in the adult hippocampus of male mice.

Nature Communications, 2024

Authors

Bielefeld P, Martirosyan A, Martín-Suárez, S, Apresyan A, Meerhoff GF, Pestana F, Poovathingal S, Reijner N, Koning W, Clement RA, Van de Veen I, Toledo E, Polzer O, Durá I, Hovhannisyan S, Nilges B, Bogdoll A, Kashikar N, Lucassen PJ, Belgard TG, Encinas JM, Holt MG, Fitzsimons CP.

Contents

Supplementary figures 1-7 and legends to supplementary figures 1-7, summarizing their content.

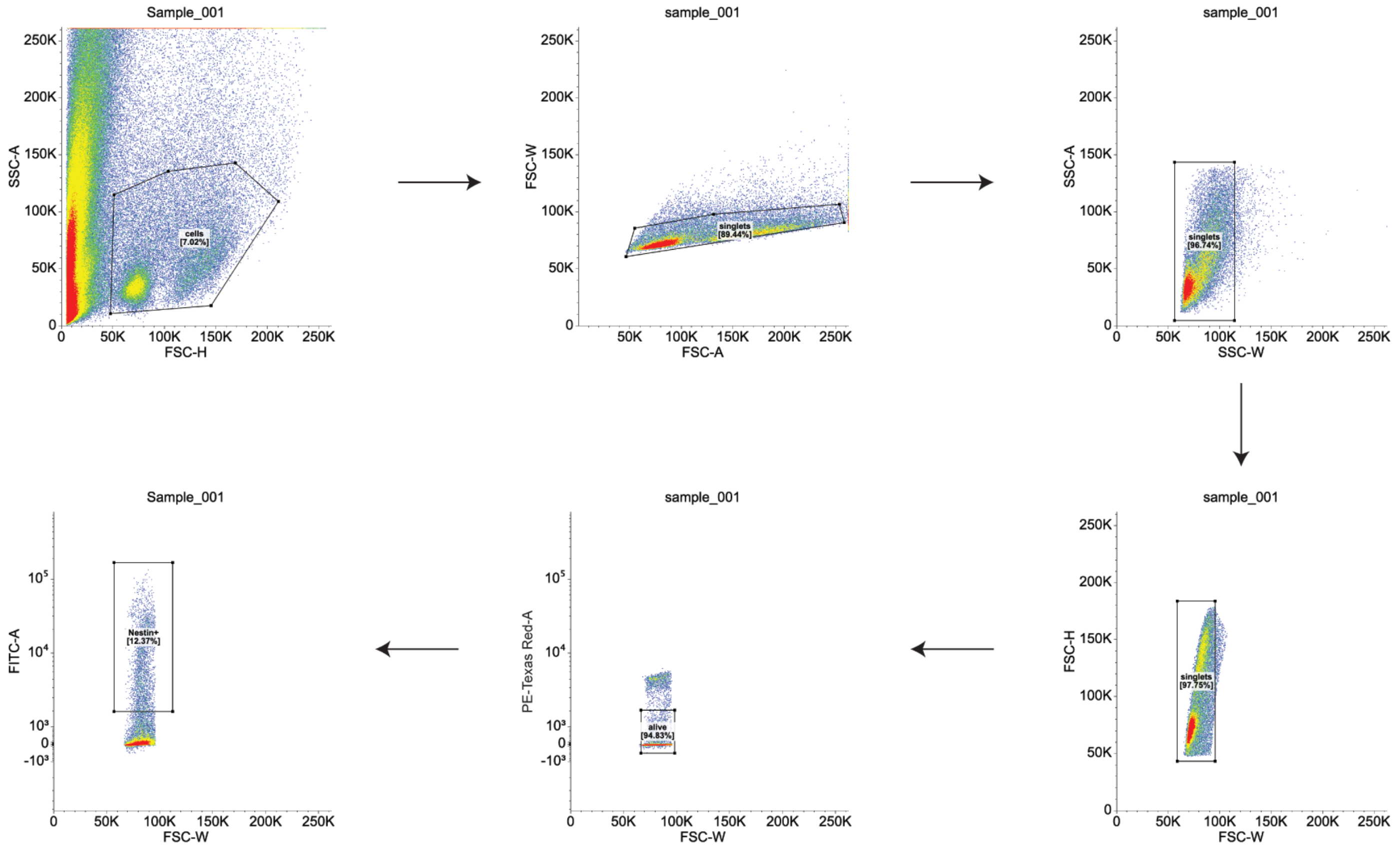

Sample_001
SSC-A
FSC-H
cells [7.02%]
sample_001
FSC-W
FSC-A
singlets [89.44%]
sample_001
SSC-A
SSC-W
singlets [96.74%]
sample_001
FSC-H
FSC-W
singlets [97.75%]
sample_001
PE-Texas Red-A
FSC-W
alive [94.83%]
Sample_001
FITC-A
FSC-W
Nestin+ [12.37%]

Legend to Supplementary Figure 1. Gating strategy used for FACS-based purification of GFP+ cells. Cells were initially selected using Forward and Side Scatter area plots (FSC-A and SSC-A) (indicated gate). To minimize the amount of small debris collected, care was taken to adjust the lower limit on the forward scatter (measure of size) axis, although the gate was left wide enough that smaller cells were still captured. Cell doublets were excluded using forward/side scatter width vs height plots (FSC-W/FSC-H and SSC-W/SSC-H). Live-dead staining using propidium iodide was used to identify viable cells, which were subsequently separated into GFP+ and GFP- populations based on fluorescence. GFP+ cells were retained for further use.

a

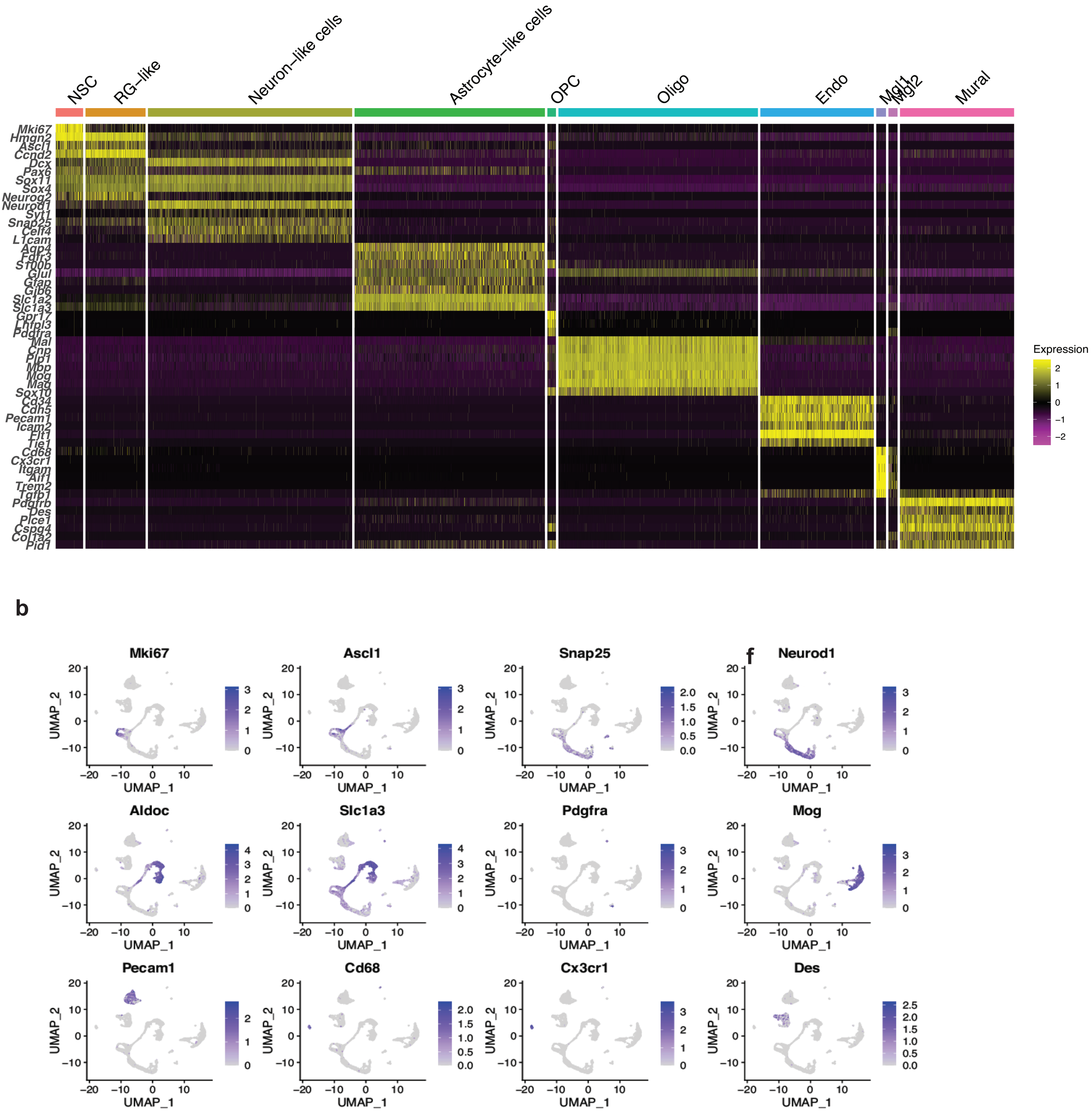
NSC
RG–like
Neuron–like cells
Astrocyte–like cells
OPC
Oligo
Endo
Mgl1
Mgl2
Mural
Mki67
Hmgn2
Ascl1
Ccnd2
Dcx
Pax6
Sox11
Sox4
Neurog2
Neurod1
Syt1
Snap25
Celf4
L1cam
Aqp4
Fgfr3
S100b
Glul
Gfap
Gjb6
Slc1a2
Slc1a3
Gpr17
Lhfpl3
Pdgfra
Mal
Cnp
Plp1
Mbp
Mog
Mag
Sox10
Cd34
Cdh5
Pecam1
Icam2
Flt1
Tie1
Cd68
Cx3cr1
Itgam
Aif1
Trem2
Tgfb1
Pdgfrb
Des
Plce1
Cspg4
Col1a2
Pid1
Expression
b
f
Mki67
Ascl1
Snap25
Neurod1
Aldoc
Slc1a3
Pdgfra
Mog
Pecam1
Cd68
Cx3cr1
Des
UMAP_1
UMAP_2

Legend to Supplementary Figure 2. Cell type assignment based on specific marker gene expression. Gene expression heatmap for higher-order cell types (columns) grouped according to the Seurat classification shown in Fig. 2a. Color-coding from Fig. 2a is retained. Magenta, low expression; yellow, high expression, ln-normalized gene expression data is shown. UMAP representations showing expression patterns of indicated marker genes across cell clusters (b), color bars indicate relative intensity of expression, SCT normalized values are shown.

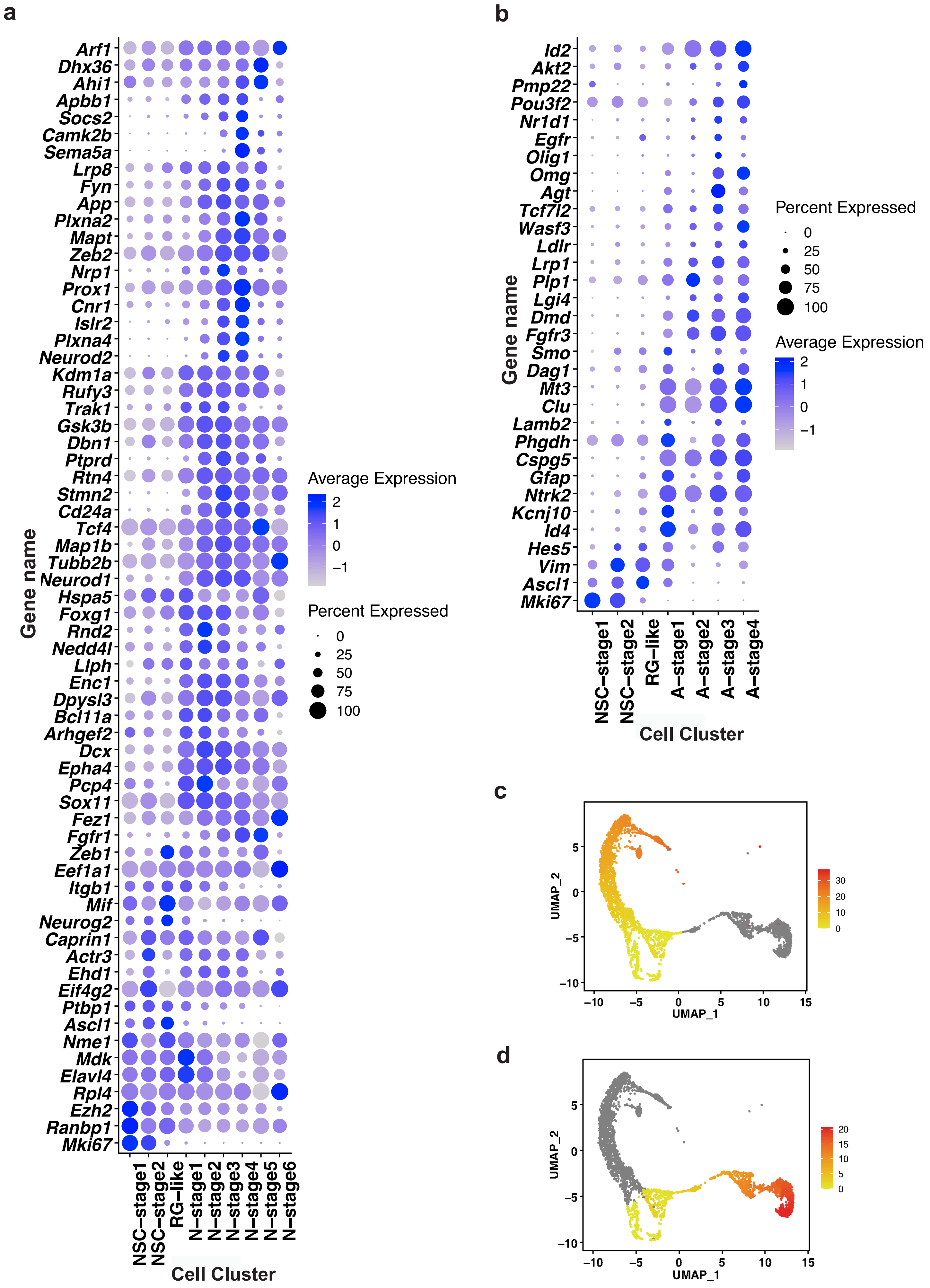
a
Arf1
Dhx36
Ahi1
Apbb1
Socs2
Camk2b
Sema5a
Lrp8
Fyn
App
Plxna2
Mapt
Zeb2
Nrp1
Prox1
Cnr1
Islr2
Plxna4
Neurod2
Kdm1a
Rufy3
Trak1
Gsk3b
Dbn1
Ptprd
Rtn4
Stmn2
Cd24a
Tcf4
Map1b
Tubb2b
Neurod1
Hspa5
Foxg1
Rnd2
Nedd4l
Llph
Enc1
Dpysl3
Bcl11a
Arhgef2
Dcx
Epha4
Pcp4
Sox11
Fez1
Fgfr1
Zeb1
Eef1a1
Itgb1
Mif
Neurog2
Caprin1
Actr3
Ehd1
Eif4g2
Ptbp1
Ascl1
Nme1
Mdk
Elavl4
Rpl4
Ezh2
Ranbp1
Mki67
Gene name
NSC−stage1
NSC−stage2
RG−like
N−stage1
N−stage2
N−stage3
N−stage4
N−stage5
N−stage6
Cell Cluster
Average Expression
2
1
0
−1
Percent Expressed
0
25
50
75
100
b
Id2
Akt2
Pmp22
Pou3f2
Nr1d1
Egfr
Olig1
Omg
Agt
Tcf7l2
Wasf3
Ldlr
Lrp1
Plp1
Lgi4
Dmd
Fgfr3
Smo
Dag1
Mt3
Clu
Lamb2
Phgdh
Cspg5
Gfap
Ntrk2
Kcnj10
Id4
Hes5
Vim
Ascl1
Mki67
Gene name
NSC−stage1
NSC−stage2
RG−like
A−stage1
A−stage2
A−stage3
A−stage4
Cell Cluster
Percent Expressed
0
25
50
75
100
Average Expression
2
1
0
−1
c
UMAP_2
UMAP_1
5
0
−5
−10
−10
−5
0
5
10
15
30
20
10
0
d
UMAP_2
UMAP_1
5
0
−5
−10
−10
−5
0
5
10
15
20
15
10
5
0

Legend to Supplementary Figure 3. Pseudotime assignment of NSC-derived cell lineages defined by expression of specific marker genes. Dot-plot graphs indicating the relative expression of marker genes in NSCs and NSC-derived neuronal (a) and astrocytic (b) populations; scaled SCT normalized data are shown. Color bars represent relative intensity of gene expression, dot size represents percentage of cells within an individual cell cluster (identity) expressing the indicated marker gene (feature). UMAP representations showing NSC-derived neuronal (c) and astrocytic lineages (d); color bars indicate calculated pseudotime distances from NSCs (yellow: minimum, red: maximum).

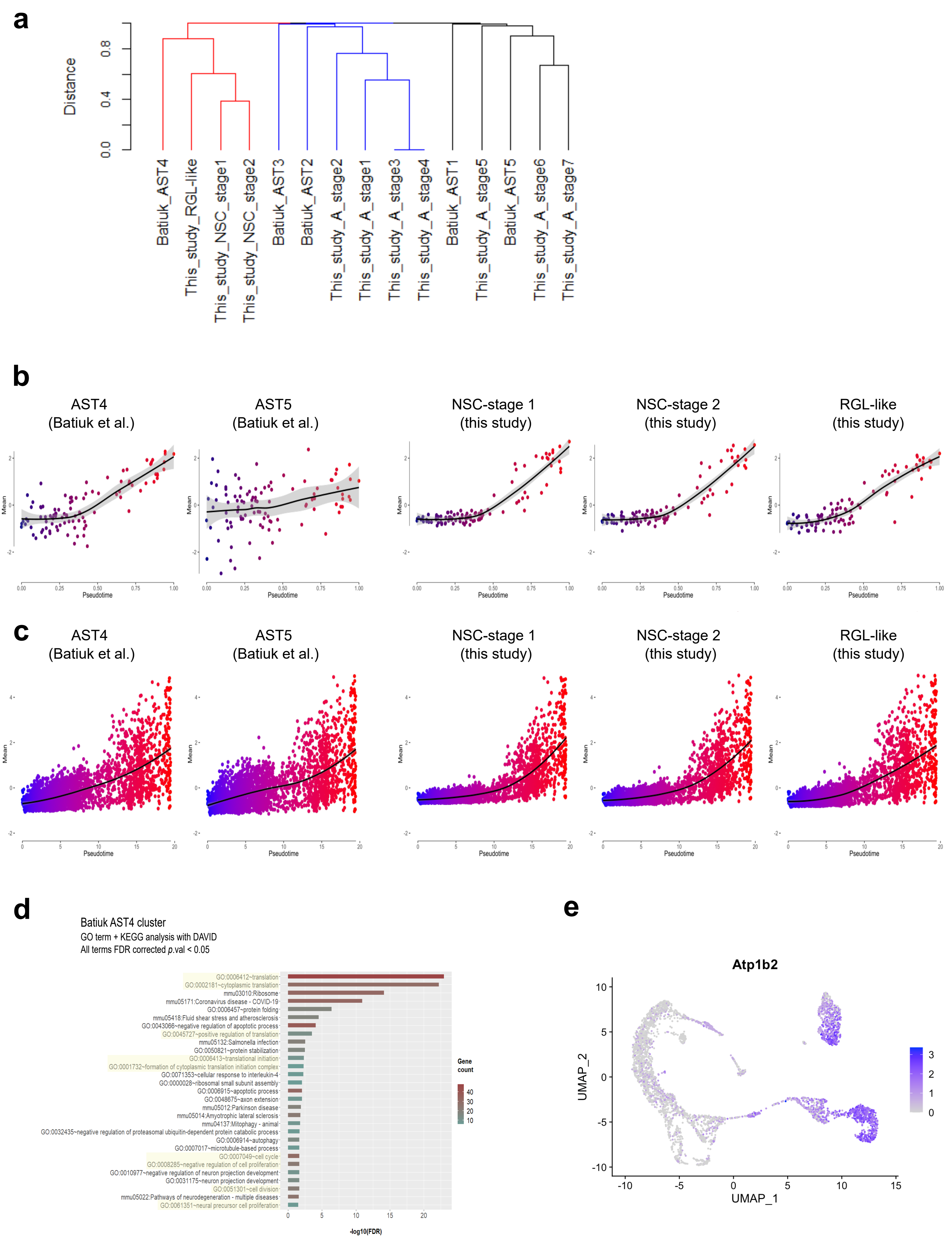
a
Distance
0.0
0.4
0.8
Batiuk_AST4
This_study_RGL-like
This_study_NSC_stage1
This_study_NSC_stage2
Batiuk_AST3
Batiuk_AST2
This_study_A_stage2
This_study_A_stage1
This_study_A_stage3
This_study_A_stage4
Batiuk_AST1
This_study_A_stage5
Batiuk_AST5
This_study_A_stage6
This_study_A_stage7
b
AST4 (Batiuk et al.)
AST5 (Batiuk et al.)
NSC-stage 1 (this study)
NSC-stage 2 (this study)
RGL-like (this study)
Mean
Pseudotime
c
AST4 (Batiuk et al.)
AST5 (Batiuk et al.)
NSC-stage 1 (this study)
NSC-stage 2 (this study)
RGL-like (this study)
Mean
Pseudotime
d
Batiuk AST4 cluster
GO term + KEGG analysis with DAVID
All terms FDR corrected p.val < 0.05
GO:0006412~translation
GO:0002181~cytoplasmic translation
mmu03010:Ribosome
mmu05171:Coronavirus disease - COVID-19
GO:0006457~protein folding
mmu05418:Fluid shear stress and atherosclerosis
GO:0043066~negative regulation of apoptotic process
GO:0045727~positive regulation of translation
mmu05132:Salmonella infection
GO:0050821~protein stabilization
GO:0006413~translational initiation
GO:0001732~formation of cytoplasmic translation initiation complex
GO:0071353~cellular response to interleukin-4
GO:0000028~ribosomal small subunit assembly
GO:0006915~apoptotic process
GO:0048675~axon extension
mmu05012:Parkinson disease
mmu05014:Amyotrophic lateral sclerosis
mmu04137:Mitophagy - animal
GO:0032435~negative regulation of proteasomal ubiquitin-dependent protein catabolic process
GO:0006914~autophagy
GO:0007017~microtubule-based process
GO:0007049~cell cycle
GO:0008285~negative regulation of cell proliferation
GO:0010977~negative regulation of neuron projection development
GO:0031175~neuron projection development
GO:0051301~cell division
mmu05022:Pathways of neurodegeneration - multiple diseases
GO:0061351~neural precursor cell proliferation
Gene count
40
30
20
10
0
5
10
15
20
-log10(FDR)
e
Atp1b2
UMAP_2
UMAP_1
10
5
0
-5
-10
15
3
2
1
0

Legend to Supplementary Figure 4. Integrated analysis of hierarchical clustering, pseudotime profiles, and functional annotations for cell populations. Dendrogram showing hierarchical clustering of putative cell populations identified in Batiuk et al.[37], or in this study (a). The labels indicate the original group names. Genes in the indicated cell populations were plotted based on the pseudotime of Shin et al.[27] (b) and of Harris et al.[29] (c). The y-axis in (b) and (c) represents the mean z-score normalization of gene expression. The genes used to identify the AST4, AST5, NSC-stage 1, NSC-stage 2 and RG-like populations were extracted from the gene lists provided with each study and the mean value was calculated. GO biological pathway analysis using DAVID for the cell cluster AST4 identified in Batiuk et al.[37] FDR: false discovery rate (d). UMAP-based visualization of NSC-derived neuronal and astrocytic lineages with *Atp1b2* expression superimposed. Each dot represents an individual cell and gene expression is indicated by color intensity (e).

a

b

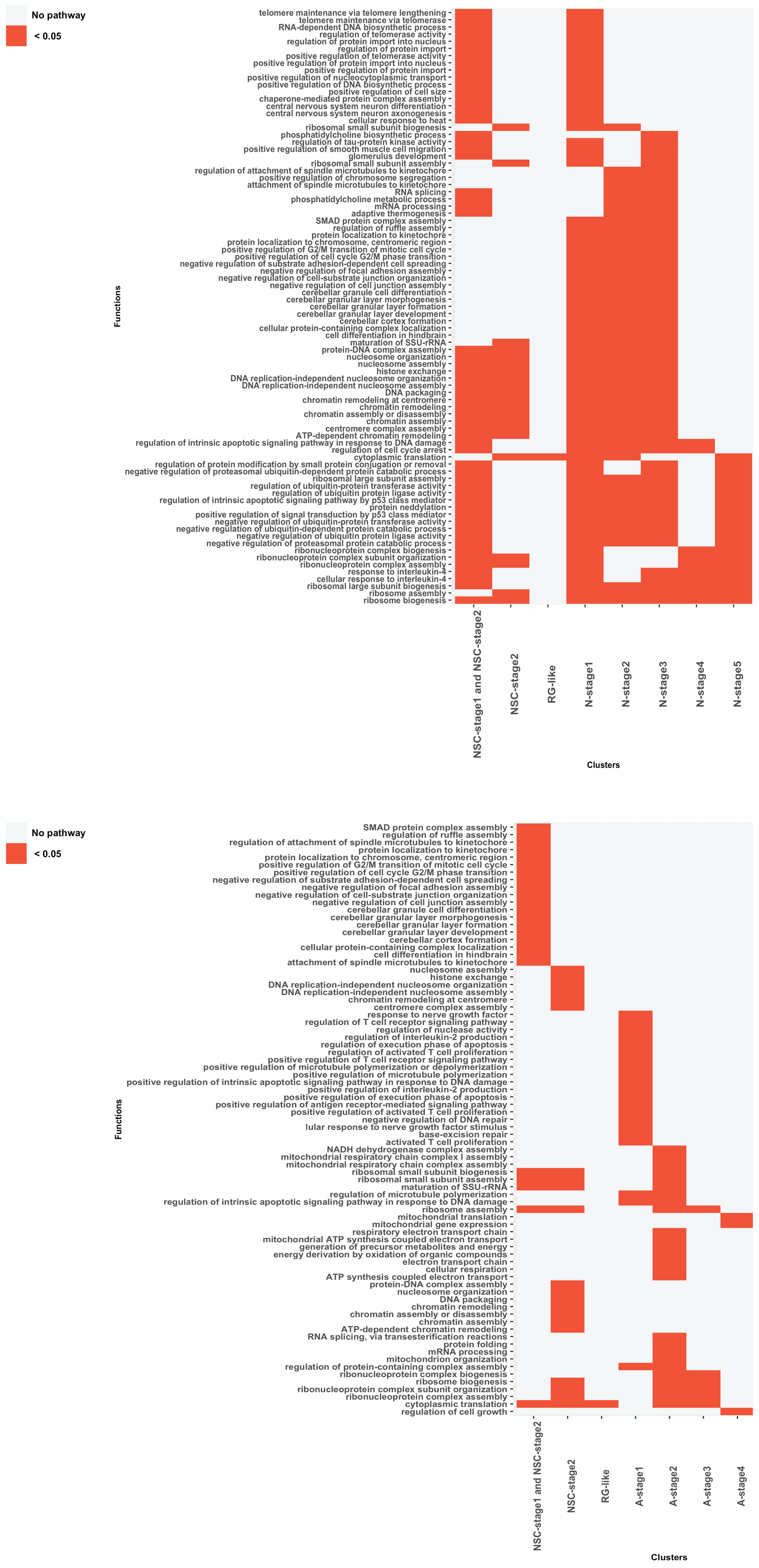

No pathway
< 0.05
telomere maintenance via telomere lengthening
telomere maintenance via telomerase
RNA-dependent DNA biosynthetic process
regulation of telomerase activity
regulation of protein import into nucleus
regulation of protein import
positive regulation of telomerase activity
positive regulation of protein import into nucleus
positive regulation of protein import
positive regulation of nucleocytoplasmic transport
positive regulation of DNA biosynthetic process
positive regulation of cell size
chaperone-mediated protein complex assembly
central nervous system neuron differentiation
central nervous system neuron axonogenesis
cellular response to heat
ribosomal small subunit biogenesis
phosphatidylcholine biosynthetic process
regulation of tau-protein kinase activity
positive regulation of smooth muscle cell migration
glomerulus development
ribosomal small subunit assembly
regulation of attachment of spindle microtubules to kinetochore
positive regulation of chromosome segregation
attachment of spindle microtubules to kinetochore
RNA splicing
phosphatidylcholine metabolic process
mRNA processing
adaptive thermogenesis
SMAD protein complex assembly
regulation of ruffle assembly
protein localization to kinetochore
protein localization to chromosome, centromeric region
positive regulation of G2/M transition of mitotic cell cycle
positive regulation of cell cycle G2/M phase transition
negative regulation of substrate adhesion-dependent cell spreading
negative regulation of focal adhesion assembly
negative regulation of cell-substrate junction organization
negative regulation of cell junction assembly
cerebellar granule cell differentiation
cerebellar granular layer morphogenesis
cerebellar granular layer formation
cerebellar granular layer development
cerebellar cortex formation
cellular protein-containing complex localization
cell differentiation in hindbrain
maturation of SSU-rRNA
protein-DNA complex assembly
nucleosome organization
nucleosome assembly
histone exchange
DNA replication-independent nucleosome organization
DNA replication-independent nucleosome assembly
DNA packaging
chromatin remodeling at centromere
chromatin remodeling
chromatin assembly or disassembly
chromatin assembly
centromere complex assembly
ATP-dependent chromatin remodeling
regulation of intrinsic apoptotic signaling pathway in response to DNA damage
regulation of cell cycle arrest
cytoplasmic translation
regulation of protein modification by small protein conjugation or removal
negative regulation of proteasomal ubiquitin-dependent protein catabolic process
ribosomal large subunit assembly
regulation of ubiquitin-protein transferase activity
regulation of ubiquitin protein ligase activity
regulation of intrinsic apoptotic signaling pathway by p53 class mediator
protein neddylation
positive regulation of signal transduction by p53 class mediator
negative regulation of ubiquitin-protein transferase activity
negative regulation of ubiquitin-dependent protein catabolic process
negative regulation of ubiquitin protein ligase activity
negative regulation of proteasomal protein catabolic process
ribonucleoprotein complex biogenesis
ribonucleoprotein complex subunit organization
ribonucleoprotein complex assembly
response to interleukin-4
cellular response to interleukin-4
ribosomal large subunit biogenesis
ribosome assembly
ribosome biogenesis
Functions
NSC-stage1 and NSC-stage2
NSC-stage2
RG-like
N-stage1
N-stage2
N-stage3
N-stage4
N-stage5
Clusters
No pathway
< 0.05
SMAD protein complex assembly
regulation of ruffle assembly
regulation of attachment of spindle microtubules to kinetochore
protein localization to kinetochore
protein localization to chromosome, centromeric region
positive regulation of G2/M transition of mitotic cell cycle
positive regulation of cell cycle G2/M phase transition
negative regulation of substrate adhesion-dependent cell spreading
negative regulation of focal adhesion assembly
negative regulation of cell-substrate junction organization
negative regulation of cell junction assembly
cerebellar granule cell differentiation
cerebellar granular layer morphogenesis
cerebellar granular layer formation
cerebellar granular layer development
cerebellar cortex formation
cellular protein-containing complex localization
cell differentiation in hindbrain
attachment of spindle microtubules to kinetochore
nucleosome assembly
histone exchange
DNA replication-independent nucleosome organization
DNA replication-independent nucleosome assembly
chromatin remodeling at centromere
centromere complex assembly
response to nerve growth factor
regulation of T cell receptor signaling pathway
regulation of nuclease activity
regulation of interleukin-2 production
regulation of execution phase of apoptosis
regulation of activated T cell proliferation
positive regulation of T cell receptor signaling pathway
positive regulation of microtubule polymerization or depolymerization
positive regulation of microtubule polymerization
positive regulation of intrinsic apoptotic signaling pathway in response to DNA damage
positive regulation of interleukin-2 production
positive regulation of execution phase of apoptosis
positive regulation of antigen receptor-mediated signaling pathway
positive regulation of activated T cell proliferation
negative regulation of DNA repair
lular response to nerve growth factor stimulus
base-excision repair
activated T cell proliferation
NADH dehydrogenase complex assembly
mitochondrial respiratory chain complex I assembly
mitochondrial respiratory chain complex assembly
ribosomal small subunit biogenesis
ribosomal small subunit assembly
maturation of SSU-rRNA
regulation of microtubule polymerization
regulation of intrinsic apoptotic signaling pathway in response to DNA damage
ribosome assembly
mitochondrial translation
mitochondrial gene expression
respiratory electron transport chain
mitochondrial ATP synthesis coupled electron transport
generation of precursor metabolites and energy
energy derivation by oxidation of organic compounds
electron transport chain
cellular respiration
ATP synthesis coupled electron transport
protein-DNA complex assembly
nucleosome organization
DNA packaging
chromatin remodeling
chromatin assembly or disassembly
chromatin assembly
ATP-dependent chromatin remodeling
RNA splicing, via transesterification reactions
protein folding
mRNA processing
mitochondrion organization
regulation of protein-containing complex assembly
ribonucleoprotein complex biogenesis
ribosome biogenesis
ribonucleoprotein complex subunit organization
ribonucleoprotein complex assembly
cytoplasmic translation
regulation of cell growth
Functions
NSC-stage1 and NSC-stage2
NSC-stage2
RG-like
A-stage1
A-stage2
A-stage3
A-stage4
Clusters

Legend to Supplementary Figure 5. GO biological pathway matrix for the differentially expressed genes in NSC-derived cell populations. Differentially over-represented (red, $p<0,05$) biological pathways found in the neuronal (a) and astrocytic (b) lineages.

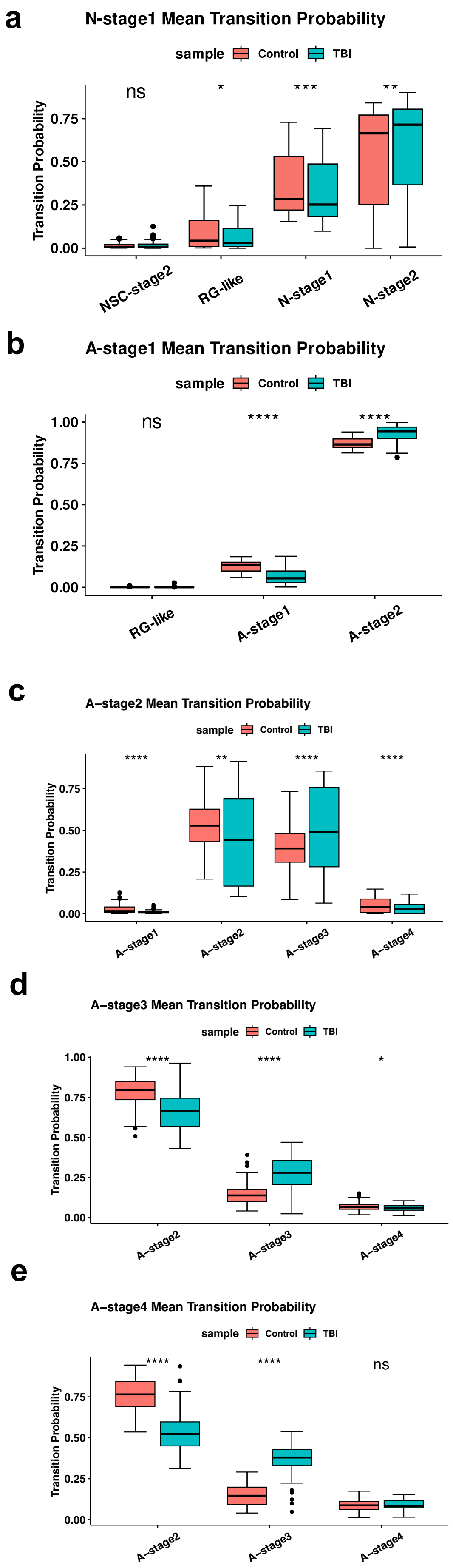
a
N-stage1 Mean Transition Probability
sample Control TBI
Transition Probability
0.00
0.25
0.50
0.75
ns
*
***
**
NSC-stage2
RG-like
N-stage1
N-stage2
b
A-stage1 Mean Transition Probability
sample Control TBI
Transition Probability
0.00
0.25
0.50
0.75
1.00
ns
****
****
RG-like
A-stage1
A-stage2
c
A–stage2 Mean Transition Probability
sample Control TBI
Transition Probability
0.00
0.25
0.50
0.75
****
**
****
****
A–stage1
A–stage2
A–stage3
A–stage4
d
A–stage3 Mean Transition Probability
sample Control TBI
Transition Probability
0.00
0.25
0.50
0.75
1.00
****
****
*
A–stage2
A–stage3
A–stage4
e
A–stage4 Mean Transition Probability
sample Control TBI
Transition Probability
0.00
0.25
0.50
0.75
****
****
ns
A–stage2
A–stage3
A–stage4

Legend to Supplementary Figure 6. RNA velocity analysis of additional cell populations. Box plots showing transition probabilities calculated for N-stage 1 cells (a), A-stage 1 cells (b), A-stage 2 cells (c), A-stage 3 cells (d) and A-stage 4 cells (e). Orange bars: Control group, cyan bars: TBI group, 5-6 animals per condition. In all panels, ns: $p > 0.05$; *p<0,05; **$p \le 0.01$; ***$p \le 0.001$; ****$p \le 0.0001$, independent two-sided non-parametric Wilcoxon test. Exact *p* values for all statistical comparisons and calculated mean transition probabilities across cell clusters are available as Supplementary data 14. Box plots show the median, first quartile (25%), third quartile (75%) and inter-quartile range. Whiskers represent data minima and maxima; dots are data points located outside the whiskers. Source data is available as Supplementary data 15.

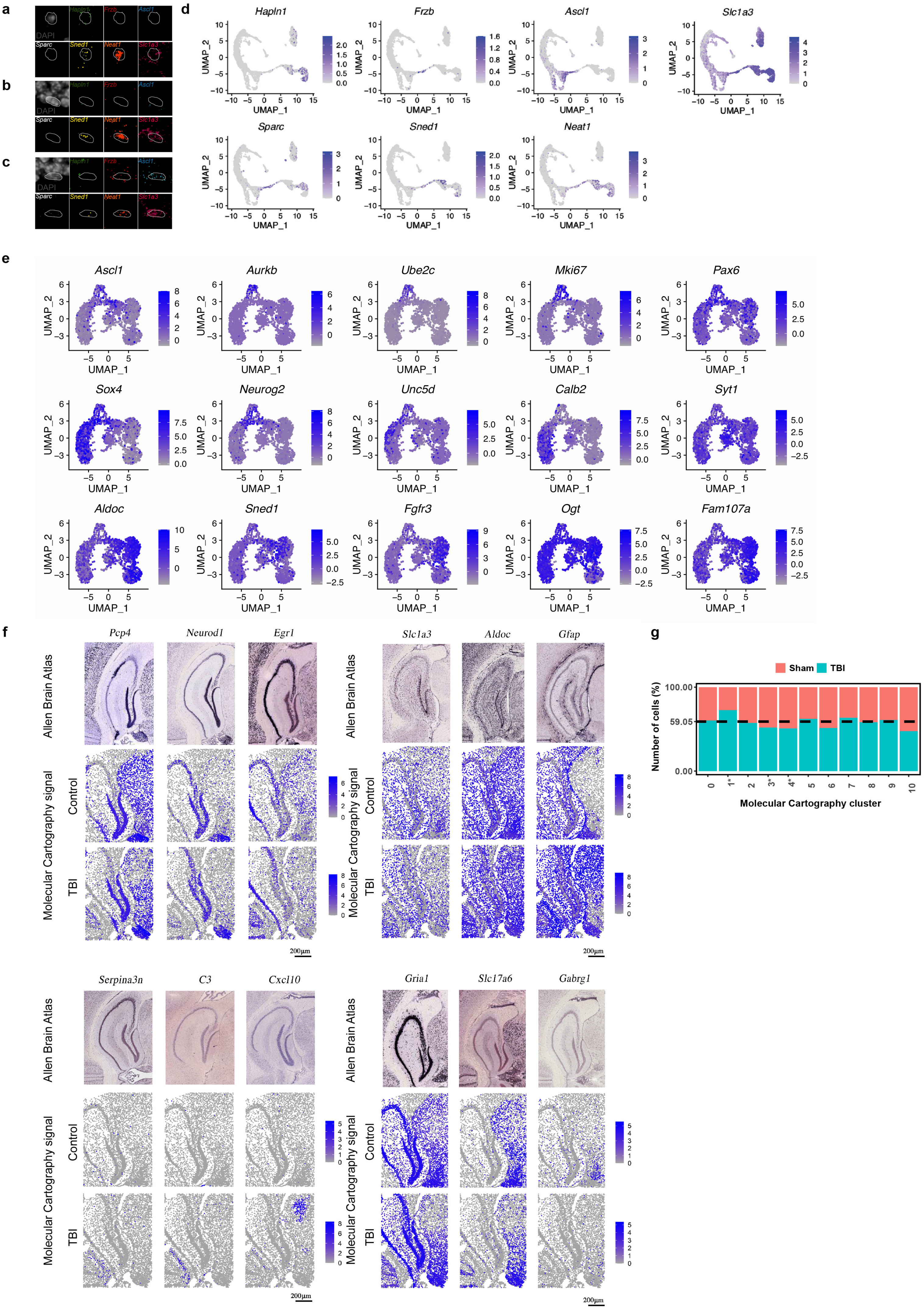
a
b
c
d
e
f
g
DAPI
Hapln1
Frzb
Ascl1
Sparc
Sned1
Neat1
Slc1a3
UMAP_1
UMAP_2
Aurkb
Ube2c
Mki67
Pax6
Sox4
Neurog2
Unc5d
Calb2
Syt1
Aldoc
Fgfr3
Ogt
Fam107a
Pcp4
Neurod1
Egr1
Gfap
Serpina3n
C3
Cxcl10
Gria1
Slc17a6
Gabrg1
Allen Brain Atlas
Molecular Cartography signal
Control
TBI
200µm
Sham
Number of cells (%)
100.00
59.05
0.00
Molecular Cartography cluster

Legend to Supplementary Figure 7. Design and validation of probes for spatial transcriptomics. Sample confocal images representative of three independent experiments of RNA scope detection of marker genes used to identify individual A-stage 4 (a), A-stage 2 (b) and A-stage 1 (c) cells in the dentate gyrus. UMAP plots showing the expression of individual gene markers used for RNAscope validation, across astrocytic cell clusters, SCT normalized values are shown (d). UMAP representations of the combined 10X and Molecular Cartography dataset, indicating expression of exemplar marker genes for the various identified cell populations (e). Sample images representative of three independent experiments showing how mRNA expression detected by 12 probes in Molecular Cartography experiments compares to that reported in the Allen Brain Atlas, colored bars indicate relative intensity of gene expression, raw counts are shown, colored dots indicate individual cells in the dentate gyrus(f). Bar plots showing the relative number of cells per identified Molecular Cartography cluster in Control or TBI samples, against their expected abundance (based on 59,05% of all cells originating from TBI samples: dashed line), *$p<0.05$ vs. expected abundance, binomial test (g). The numbers of cells per cluster and experimental condition, and the statistical analyses performed are given in Supplementary data 11, including exact *p* values for all statistical comparisons.
