# Supplementary material for: Traumatic brain injury promotes neurogenesis at the cost of astrogliogenesis in the adult hippocampus of male mice
Source: Nat Commun. 2024 Jun 18;15:5222. doi: 10.1038/s41467-024-49299-6 (PMC11189490; doi:10.1038/s41467-024-49299-6)
Supplement: Supplementary file 3 — Description of Additional Supplementary Files [file 41467_2024_49299_MOESM3_ESM.docx]

**Legends for supplementary data files associated to:**

**Traumatic brain injury promotes neurogenesis at the cost of astrogliogenesis in the adult hippocampus of male mice.**

**Nature Communications, 2024**

**Authors**

Bielefeld P, Martirosyan A, Martín-Suárez, S, Apresyan A, Meerhoff GF, Pestana F, Poovathingal S, Reijner N, Koning W, Clement RA, Van de Veen I, Toledo E, Polzer O, Durá I, Hovhannisyan S, Nilges B, Bogdoll A, Kashikar N, Lucassen PJ, Belgard TG, Encinas JM, Holt MG, Fitzsimons CP.

**Supplementary data 1. Number of cells per major cell population in the dentate gyrus, based on clustering of the single cell data.**

**Supplementary data 2. Number of cells per cell subpopulation in the dentate gyrus, based on clustering of the single cell data.**

**Supplementary data 3. Marker genes defining cell clusters identified in the 10X Genomics scRNA-seq dataset.** SCT normalized data.

**Supplementary data 4. Full set of GO and KEGG terms linked to cell subtype specific markers.**

**Supplementary data 5. Selected set of GO and KEGG terms linked to cell subtype specific markers.**

**Supplementary data 6. Average transition probabilities per cell predicted using RNA velocity analysis.**

**Supplementary data 7. Differentially expressed genes per cell population in Control vs. TBI groups.** Genes were identified using a MAST test on SCT normalized data.

**Supplementary data 8. Full set of GO and KEGG terms linked to up or down-regulated genes after TBI.**

GO and KEGG terms included: log fold change > 0.2 and adjusted *p* value < 0.05.

**Supplementary data 9. Selected set of GO and KEGG terms linked to up/down-regulated genes after TBI.** GO and KEGG terms included: log fold change > 0.2 and adjusted *p* value < 0.05.

**Supplementary data 10. Overview of targets in the Molecular Cartography spatial transcriptomics experiments.**

**Supplementary data 11. Cell segmentation QuPath parameters used to define cell boundaries in the Molecular Cartography experiments.**

**Supplementary data 12. Parameters used to run RNA velocity analysis per cell population.**

**Supplementary data 13. Number of cells per cluster and experimental condition, binomial test results.**

**Supplementary data 14. Mean transition probability across cells clusters**

**Supplementary data 15. Source data for Figure 3 and Supplementary Figure 6**
